# Supplementary figures and images for: Development of the PD9-9 Monoclonal Antibody for Identifying Porcine Bone Marrow-Derived Dendritic Cells
Source: Life (Basel). 2024 Aug 23;14(9):1054. doi: 10.3390/life14091054 (PMC11433566; doi:10.3390/life14091054)

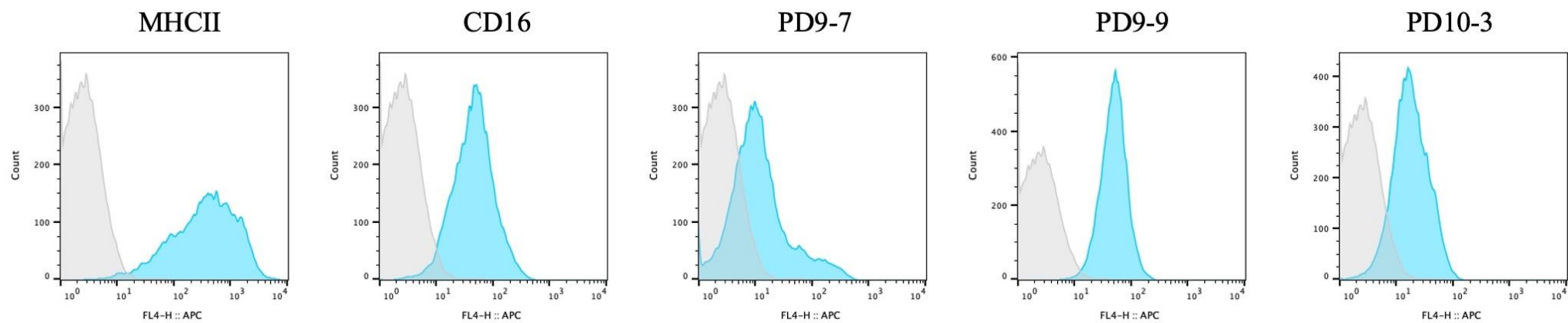

Figure S1: Monoclonal antibodies (mAbs) that react to porcine dendritic cells.

Supplement: Supplementary file 1 [file life-14-01054-s001.zip › life-3112846-supplementary.pdf]
